# Bacterial guilds, not genus-level taxa, mediate the protective effects of time-restricted feeding against high-fat diet–induced obesity in mice

**DOI:** 10.1093/ismeco/ycaf127

**Published:** 2025-10-11

**Authors:** Shreya Ghosh, Yue Li, Xin Yang, Guojun Wu, Chenhong Zhang, Liping Zhao

**Affiliations:** State Key Laboratory of Microbial Metabolism, Joint International Research Laboratory of Metabolic & Developmental Sciences, School of Life Sciences and Biotechnology, Shanghai Jiao Tong University, Shanghai 200240, China; Department of Biochemistry and Microbiology, School of Environmental and Biological Sciences, Rutgers, The State University of New Jersey, New Brunswick, NJ 08901, United States; State Key Laboratory of Microbial Metabolism, Joint International Research Laboratory of Metabolic & Developmental Sciences, School of Life Sciences and Biotechnology, Shanghai Jiao Tong University, Shanghai 200240, China; State Key Laboratory of Microbial Metabolism, Joint International Research Laboratory of Metabolic & Developmental Sciences, School of Life Sciences and Biotechnology, Shanghai Jiao Tong University, Shanghai 200240, China; Department of Biochemistry and Microbiology, School of Environmental and Biological Sciences, Rutgers, The State University of New Jersey, New Brunswick, NJ 08901, United States; New Jersey Institute for Food, Nutrition, and Health, Rutgers, The State University of Jersey, New Brunswick, NJ 08901, United States; State Key Laboratory of Microbial Metabolism, Joint International Research Laboratory of Metabolic & Developmental Sciences, School of Life Sciences and Biotechnology, Shanghai Jiao Tong University, Shanghai 200240, China; State Key Laboratory of Microbial Metabolism, Joint International Research Laboratory of Metabolic & Developmental Sciences, School of Life Sciences and Biotechnology, Shanghai Jiao Tong University, Shanghai 200240, China; Department of Biochemistry and Microbiology, School of Environmental and Biological Sciences, Rutgers, The State University of New Jersey, New Brunswick, NJ 08901, United States; New Jersey Institute for Food, Nutrition, and Health, Rutgers, The State University of Jersey, New Brunswick, NJ 08901, United States

**Keywords:** gut microbiota, bacterial guilds, time-restricted feeding, glucose intolerance, diurnal microbial oscillation

## Abstract

The gut microbiota functions as a complex adaptive system where microbes form structural modules known as “guilds.” Each guild comprises taxonomically distinct microbes that work together as cohesive functional units, contributing to overall system function. Traditional taxon-based microbiome analyses often yield inconsistent associations with disease, limiting mechanistic insights. To address this, we compared guild-based and taxon-based approaches using datasets from a time-restricted feeding (TRF) study in mice. C57BL/6 J male mice were assigned to *ad libitum* feeding or TRF groups, with metabolic parameters and gut microbiota composition assessed over 12 weeks. Isocaloric TRF improved glucose tolerance and reduced weight gain in high-fat diet (HFD)–fed mice while maintaining metabolic stability in normal-fat diet–fed mice. To examine microbial contributions, 293 prevalent amplicon sequence variants (ASVs) from the 16S rRNA gene’s V3–V4 regions were clustered into 34 co-abundance groups (CAGs), representing potential microbial guilds and accounting for 96% of the total sequence abundance. By contrast, the taxon-based approach classified 660 ASVs into 126 genera, capturing only 78% of the total sequence abundance while omitting 22% of sequences representing novel microbes. The 34 CAGs preserved community-level information more effectively than the 66 prevalent genera, as demonstrated by Procrustes analysis. Five CAGs correlated with improved metabolic phenotype under TRF, including unclassifiable ASVs. Notably, two key CAGs exhibited conserved diurnal rhythmicity under TRF. In contrast, ASVs within putative health-relevant genera displayed opposing TRF responses. This study underscores microbial guilds as key mediators of TRF’s metabolic benefits and emphasizes the need to recalibrate taxon-based microbiome analysis biomarker discovery.

## Introduction

Traditional taxon-based analyses are valuable for cataloging microbial composition but often fall short of capturing the functional complexity of microbial ecosystems. These methods rely on reference databases, which can lead to the exclusion of novel or unclassified microbial sequences and provide limited insights into the ecological roles and interactions within communities. By focusing solely on taxonomic classifications, important functional diversity, and microbial dynamics within taxa are often overlooked. As microbiome datasets become increasingly extensive and complex, the limitations of taxon-based methods become more pronounced, potentially obscuring key ecological interactions and functional relationships. For example, studies have shown conflicting results regarding the response of the genus *Lactobacillus* to time-restricted feeding (TRF), with some reporting a decrease and others an increase in abundance [1, 2]. Such inconsistencies, along with the exclusion of unclassifiable sequences, undermine the foundation needed to identify active microbes crucial for further mechanistic studies.

To address these limitations comprehensively, we developed a guild-based approach that clusters amplicon sequence variants (ASVs) into co-abundance groups (CAGs) based on shared ecological behavior rather than relying on taxonomic classifications alone [3–5]. This approach conceptualizes the gut microbiota as a complex adaptive system where members, as represented by individual ASVs, interact and form coherent functional modules, or “guilds,” providing a more granular and ecologically relevant understanding of microbial dynamics [4, 6–8].

To evaluate the divergence with taxon-based analysis, we apply this guild-based approach on the same datasets produced from a study investigating the effects of TRF on the gut microbiota. TRF, which restricts food intake to an 8–12-h window during the active phase of mice, has been shown to mitigate weight gain and metabolic dysfunction induced by a high-fat diet (HFD) [9, 10]. Despite these benefits, the mechanisms through which TRF improves metabolic health remain partially understood. Recent studies suggest that TRF influences the diurnal oscillation of the gut microbiota, potentially mediating the diet’s health effects. Diurnal oscillation, primarily driven by diet and feeding patterns, is evident in mice fed a normal chow diet [1, 11, 12], with increased abundance of Firmicutes during active phases and decreased abundance during rest phases [1]. However, this rhythmicity is disrupted in mice with *ad libitum* HFD access, where Firmicutes dominate regardless of the time of day [1]. Although some studies suggest that TRF can modify the abundance of major phyla like Bacteroidetes and Firmicutes [13], others report no changes at the phylum level [1] but rather at lower taxa, such as the *Lactobacillus* genus, which loses its diurnal pattern in unrestricted HFD conditions [1]. Additionally, taxa like *Ruminococcus* and Christensenellaceae have been noted to increase under TRF [14]. These findings highlight that various taxon-based microbiome features are associated with TRF’s beneficial effects.

In this study, we investigate the effects of isocaloric TRF on the gut microbiota, glucose tolerance, and diurnal oscillation in mice fed an HFD with a fiber-matched low-fat control diet. By controlling for both caloric intake and dietary fiber, we address key limitations in previous studies that often overlooked these critical factors for gut microbiota and metabolic health. Our analysis focuses on whether TRF conserves the gut microbiota’s natural diurnal rhythm in HFD-fed mice and whether this conservation is linked to improved metabolic health. Importantly, we employ a guild-based analysis to capture dynamic microbial responses to TRF, overcoming the limitations of genus-based methods, which tend to exclude novel, yet significant, microbial members and often generate misleading associations with metabolic outcomes. By shifting from traditional taxonomic classifications to an ecologically meaningful analysis, we identified specific microbial guilds that respond to TRF and are associated with positive metabolic changes. These findings provide fresh insights into the development of novel health strategies targeting functional guilds, highlighting the necessity of recalibrating taxon-based signatures for microbiome biomarker discovery.

## Materials and methods

### Animal experiment and sample collection

All animal experiments were approved by the Institutional Animal Care and Use Committee (IACUC) of the School of Life Sciences and Biotechnology, Shanghai Jiao Tong University (No. 2018035).

**Figure 1:**
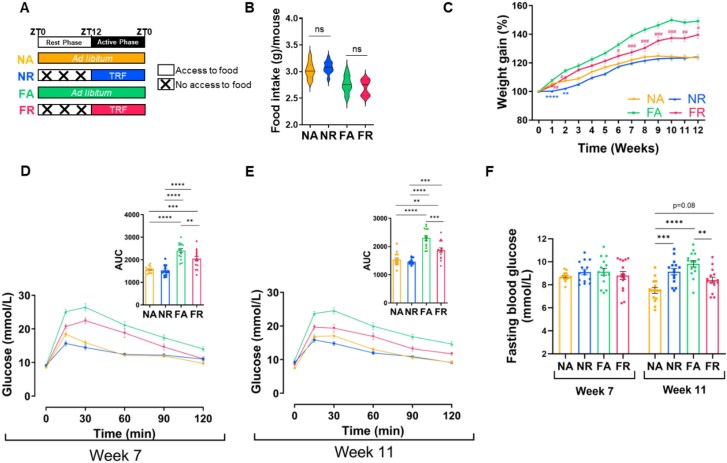
Time-restricted feeding (TRF) mitigated the HFD-induced adverse metabolic effects in mice. (A) Outline of study design indicating the different diet and feeding regimes. NA and FA had *ad libitum* access to food. NR and FR had time-restricted access to food from ZT12 to ZT0. NA and NR were fed a normal-fat diet (NFD), while FA and FR were fed a high-fat diet (HFD). The colored bar indicates access to food. (B) Average daily food intake during the study period is represented as a violin plot; the solid line indicates the median, while the dotted line indicates the interquartile range. Analyzed by unpaired *t*-test (two-tailed) for each diet. (C) Percent body weight gain over time for each group. Data expressed as mean ± SEM and analyzed by one-way ANOVA followed by Tukey’s test at each time point ^*^P < .05, ^**^P < .01, ^***^P < .001, ^****^P < .0001 (compared to NA); ^#^*P* < .05, ^##^*P* < .01, ^###^*P* < .001, ^####^*P* < .0001 (compared to FA). *n* = 15/group except NR, *n* = 14 for Week 12. The color of the symbols indicates the group that is significantly different at the specific time point. For improved visualization, only comparisons between NA vs. NR and FA vs. FR are shown. (D) Oral glucose tolerance test (OGTT) after 7 weeks (*n* = 15/group) and (E) 11 weeks of study duration with the calculated area under the curve (AUC) of blood glucose (*n* = 15/group, except NR, *n* = 14). (F) Fasting blood glucose. (D–F) Data expressed as mean ± SEM and analyzed by one-way ANOVA followed by Tukey’s test at each time point; ^*^P < .05, ^**^P < .01, ^***^P < .001, ^****^P < .0001.

Specific pathogen-free (SPF), 5-week-old male mice (C57BL/6 J) were purchased from SLAC Inc. (Shanghai, China) and acclimated at the animal center, Shanghai Jiao Tong University, for 3 weeks before the start of the experiment. Mice were housed under a 12-h light:12-h dark cycle (lights on from 7 a.m. to 7 p.m.; ZT0–ZT12) and at a constant temperature of 22°C ± 3°C. During acclimation, mice had *ad libitum* access to a normal-fat diet (NFD; D12450J Research Diets, NJ, USA; Table S1) and autoclaved water. After acclimation, mice were randomly assigned to one of four groups (15 mice per group, 3 mice per cage) for 12 weeks (Fig. 1A) (i) NFD *ad libitum* (NA), (ii) NFD with 12 h TRF (NR), (iii) HFD *ad libitum* (FA; HFD, D12492, Research Diets; Table S1), and (iv) HFD with 12 h TRF (FR). NFD and HFD contained similar amounts of dietary fiber to control for its confounding effect on the diurnal behavior of the gut microbiota. Mice TRF groups (NR and FR) were transferred daily between feeding cages (with food and water) and fasting cages (with only water). Mice with *ad libitum* access were also transferred between cages to control for the effects of cage transfer [1]. The body weight of the individual mice and food intake per cage were measured twice weekly at the same time every week. At the end of the study period, mice were fasted for 6 h (ZT0–ZT6) before sacrifice. Serum and tissue samples were collected and stored at −80°C until further analysis. Fresh fecal samples (two to three fecal pellets/mice) were collected from the mice individually and stored at −80°C until DNA extraction. Indirect calorimetry was performed on a subset of mice (*n* = 5/group) using a Comprehensive Lab Animal Monitoring System (CLAMS, Columbus Instruments) at the Shanghai Model Organisms Center, Inc. (Shanghai, China). Mice were placed individually in the metabolic chambers for three consecutive days including 24 h of acclimation with conditions maintained as in home cages.

### Oral glucose tolerance test

Baseline blood glucose was measured after a 6-h fast (ZT0–ZT6) using a blood glucose meter (ACCUCHEK® Performa, Roche, USA). Glucose (2.0 g/kg body weight) was administered via oral gavage and blood glucose levels were measured at 15, 30, 60, 90, and 120 min postadministration using blood collected from the tail vein.

### Microbial 16S rRNA gene V3–V4 region sequencing and data analysis

Microbial DNA was extracted from the fecal samples collected from the individual mice at baseline (before the feeding regime) samples and after 12 weeks of the feeding according to a method described previously [15]. To account for diurnal variation, samples were collected at the same time (ZT1, 8 a.m.). In Week 11, fecal samples were collected every 6 h over 48 h (*n* = 3–5/group). The 16S rRNA gene’s V3–V4 region was sequenced using the MiSeq sequencing platform (Illumina Inc., USA), with library preparation following a modified version of the manufacturer’s instructions, as described previously [16].

The 16S rRNA gene amplicon sequence data were processed and analyzed on the QIIME2 software (v2019.7) [17]. Raw sequence data were demultiplexed and then denoised with the DADA2 pipeline (q2-dada2 plugin) [18] to obtain the ASV frequency table. Alpha diversity metrics (observed ASVs and Shannon’s index), beta diversity metric (Bray–Curtis dissimilarity), and principal coordinate analysis (PCoA) were performed using the “core-metrics-phylogenetic” plugin after rarefying the samples to 18 000 sequences per sample for baseline and Week 12 data. R “ggplot2” package and MATLAB (R2024b) were used to plot the graphs. Alpha diversity indices were compared with Mann–Whitney tests. Statistical significance between the groups was assessed by permutational multivariate analysis of variance (PERMANOVA) with 9999 permutations, and *P*-values obtained were adjusted with the Benjamini–Hochberg correction method [19]. Taxonomic assignment for ASVs was performed via the q2-feature-classifier [20] using the SILVA 132 16S rRNA gene database [21]. For Week 11 data, samples were rarefied to 18 000 reads for subsequent analysis. Beta diversity metric (Bray–Curtis dissimilarity) and PCoA analysis were computed as mentioned earlier in this section. Diurnal patterns in PCs/CAGs/ASVs were analyzed using empirical JTK_CYCLE [22] with a set period of 24 h [12].

### Microbial co-abundance group network analysis

Correlation coefficients between ASVs present in over 25% of the samples were calculated using Bland and Altman’s method for repeated observations [23, 24] after transforming the ASV table using robust CLR [25]. Next, the matrix was converted into a distance matrix (1-correlation coefficient) and subsequently clustered using Ward’s hierarchical clustering to build a tree. Then, PERMANOVA (9999 permutations) was applied consecutively from the top of the tree to determine distinct clades (CAGs) with a significance cutoff of *P* < .001 [4, 26]. The CAG abundance was obtained as the sum of all the ASVs belonging to that CAG [4]. The CAG network was visualized in Cytoscape v3.10.0 [27].

### Statistical analysis

Statistical analyses were conducted using Graphpad Prism 10 software and R (version 4.2.2) with the “ComplexHeatmap” [28] and “ggplot2” packages. One-way ANOVA with Tukey *post hoc* test was used for physiological and biochemical data, with significance set at *P*-value <.05. A linear mixed-effects model was implemented using the “lme4” package to assess the impact of cage as a random effect on phenotype data [29]. The data obtained from the metabolic cage were analyzed with CalR software [30]. PCoA and PERMANOVA (9999 permutations) were performed in QIIME2 to assess Bray–Curtis dissimilarity for CAGs (genera). Procrustes analysis, using QIIME2’s “procrustes-analysis” plugin and R’s “vegan” package PROTEST, assessed concordance between CAG (genus) and ASV. Boruta, a Random Forest–based feature selection method, was used to identify the key CAGs capable of discriminating between the groups [31]. The receiver operating characteristic (ROC) curve was generated using the “evalm” function from the R package “MLeval” for validation of the CAG-based classification. Spearman correlation analysis, followed by FDR correction using Benjamini and Hochberg, was used to determine correlations between CAGs (genera) and metabolic phenotypes [19].

## Results

### Time-restricted feeding reduced body weight gain and glucose intolerance compared to *ad libitum* high-fat diet feeding, despite isocaloric intake

To assess if TRF influenced the observed metabolic outcomes independently of calorie intake, 8-week-old male C57BL/6 J mice were randomly assigned to one of the following four groups (Fig. 1A): (i) NA (*ad libitum* NFD), (ii) NR (TRF with NFD, meaning access to food was limited to the active phase, i.e. ZT12-ZT0, (iii) FA (ad libitum HFD), and (iv) FR (TRF with HFD). No significant differences in baseline body weight were observed among the four groups, after adjusting for potential cage effects (*F* = 0.083, *P* = .969). Calorie intake remained the same for both treatments on the same diet (Fig. 1B).

TRF did not impact the weight gain in NFD-fed mice. FR mice gained significantly less weight than FA mice, despite similar calorie intake (Fig. 1C). To determine TRF's impact on glucose tolerance, an oral glucose tolerance test (OGTT) was performed after 7 and 11 weeks of intervention (Fig. 1D–F). The FR mice demonstrated a significantly faster glucose clearance rate compared to the FA mice during both OGTTs. However, TRF did not improve glucose tolerance in NFD-fed mice, with both groups showing similar glucose clearance during OGTTs. Furthermore, fasting blood glucose in the FR mice was significantly lower than in the FA group during the OGTT conducted after 11 weeks of intervention. Interestingly, fasting blood glucose levels measured after 11 weeks of intervention were comparable between the FR and NA mice. In contrast, NA mice had significantly lower fasting blood glucose than NR mice after 11 weeks, but no significant difference in glucose tolerance was observed. TRF-induced changes remained consistent after accounting for potential cage effects (Table S2).

Overall, TRF improved the metabolic outcomes in mice fed with isocaloric HFD.

### Time-restricted feeding changed the overall gut microbiota structure only in mice fed with high-fat diet

To investigate how the gut microbiota responded to TRF, we sequenced the V3–V4 region of the 16S rRNA gene from the DNA extracted from fecal samples collected before (baseline) and after 12 weeks of dietary intervention. We obtained 4 864 921 high-quality reads (average 41 228 ± 5562 reads per sample). The sequencing depth was rarefied to 18 000 reads per sample, 1111 ASVs remained for further analysis.

**Figure 2:**
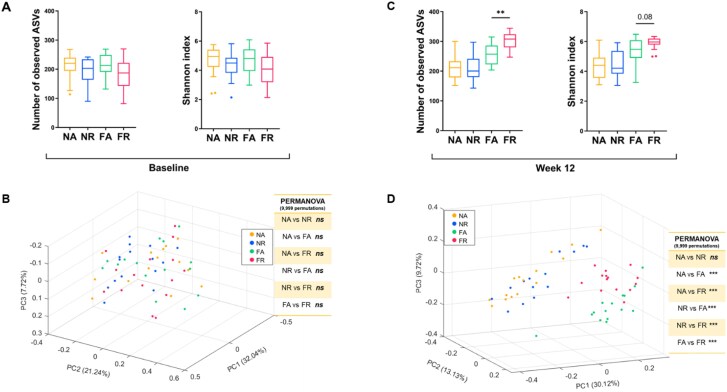
TRF altered the overall gut microbiota structure in HFD-fed mice at the ASV level. Alpha diversity was measured by the number of observed amplicon sequence variants (ASVs) and Shannon index at (A) baseline and (C) after 12 weeks of TRF. Data expressed as mean ± SEM, and the Mann–Whitney test was used to compare the effect of a difference in feeding regime in each diet. Principal coordinate analysis (PCoA) plot of the gut microbiota structure based on the Bray–Curtis dissimilarity metric with corresponding PERMANOVA comparisons (9999 permutations) and *P*-values adjusted with Benjamini–Hochberg correction for multiple group comparison at (B) baseline and (D) after 12 weeks of TRF.

The overall gut microbiota structure did not differ between the groups at baseline (Fig. 2A and B) and was not influenced by cage effects (Fig. S1 and Table S3). After 12 weeks of intervention, FR mice exhibited increased gut microbiota diversity (Shannon index) and richness (number of observed ASVs) compared to FA mice (Fig. 2C). However, alpha diversity measures did not differ between the NR and NA groups. PCoA and PERMANOVA based on the Bray–Curtis dissimilarity metric of all the 1111 ASVs showed significant differences in the overall gut microbiota structure between the FR and FA groups but not between the NR and NA groups (Fig. 2D). The TRF effect (*F* > 14) exceeded the cage effect (*F* < 3), indicating that TRF explained more variation in microbiome composition (Table S3). This indicates that TRF altered the overall structure of the gut microbiota in HFD-fed mice, consistent with the improvements observed in metabolic outcomes and not confounded by cage effects.

**Figure 3:**
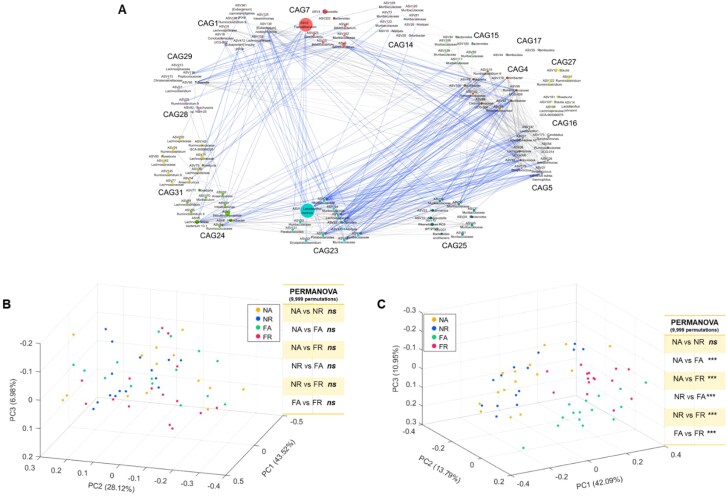
TRF altered the overall gut microbiota structure at the co-abundance group (CAG) level in HFD-fed mice (A) Microbial interaction network formed with prevalent ASVs from the four groups using baseline and Week 12 abundance data. The network plot displayed the interactions between the different CAGs. Node size represents the mean abundance of ASV. The edges between the nodes indicate correlation (gray = positive, blue = negative), with the width of the edge corresponding to the magnitude of the correlation. Correlations with absolute values > 0.5 and CAGs with mean abundance >1% are shown here for improved visualization. Principal-coordinate analysis (PCoA) analysis was performed using the relative abundance profiles for the 34 co-abundance groups (CAGs) based on the Bray–Curtis dissimilarity metric at (B) baseline and (C) after 12 weeks of the TRF regime.

To identify TRF-responsive guilds, we constructed a co-abundance network with 293 prevalent ASVs found in over 25% of the samples, accounting for ~96% of the total reads (Fig. 3A and Table S4). These 293 prevalent ASVs were clustered into 34 CAGs. PCoA performed on the relative abundance profiles for the 34 CAGs using the Bray–Curtis dissimilarity metric showed that all four groups had similar gut microbiota structures at the CAG level (Fig. 3B). After 12 weeks of dietary intervention, FR and FA groups formed separate clusters in the PCoA plot, while mice from NR and NA groups were indistinguishable (Fig. 3C). Additionally, PERMANOVA analysis showed significant differences between FR and FA groups, but not between NR and NA groups, after 12 weeks of intervention (Fig. 3C).

In summary, both the CAG level data (34 CAGs) and the ASV level (1111 ASVs) showed that TRF altered the overall gut microbiota structure in HFD-fed mice, consistent with the improved metabolic phenotype observed in these mice.

### Time-restricted feeding-responding co-abundance groups in gut microbiota were associated with improved metabolic outcomes in high-fat diet–fed mice

**Figure 4:**
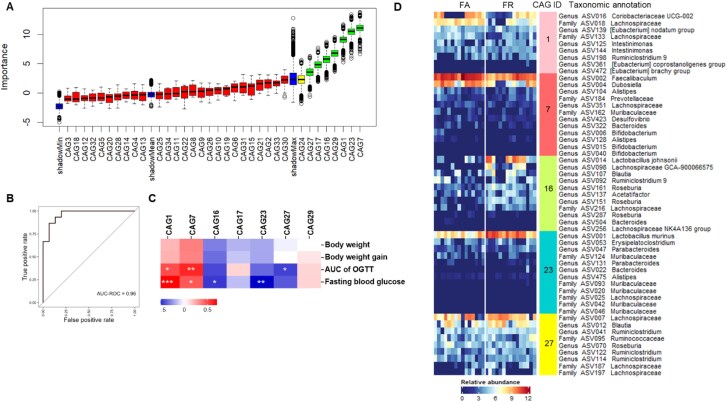
TRF-responding CAGs were associated with improved metabolic outcomes in HFD-fed mice. (A) Boxplot of the importance score (*Z* score) of the CAGs identified by Boruta for differentiating between FA and FR after 12 weeks of intervention. The boxplots in “green” were identified as key variables capable of discriminating between FA and FR, whereas the boxplots in “red” were found as nondiscriminatory. The boxplots in “blue” refer to the minimum, mean, and maximum *Z* scores of a shadow variable. (B) The area under the ROC curve for the CAG-based classification of the FA and FR group (AUC = 0.960); ROC, receiver operating characteristic. (C) Heatmap of Spearman’s correlation (with FDR correction) between the relative abundance of the discriminating CAGs identified by Boruta and the metabolic parameters related to glucose and lipid metabolism. ^*^P < .05, ^**^P < .01, ^***^P < .001. (D) Heatmap of relative abundance of the ASVs (log2-transformed) that form the key CAGs that showed significant correlation with the host metabolic parameters.

TRF differentially altered the abundance of CAGs in the FR and FA groups. Using Boruta, a Random Forest–based feature selection algorithm, we identified seven TRF-responding CAGs that distinguished between FR and FA mice after 12 weeks of intervention (Fig. 4A). The area under the ROC curve (AUC-ROC = 0.96) (Fig. 4B) showed excellent performance of the classification model. Five of the seven TRF-responding CAGs were identified as health-relevant as they significantly correlated with fasting blood glucose and AUC-OGTT (Fig. 4C). Among these, CAG16, CAG23, and CAG27 were significantly more abundant in FR mice compared with FA mice (Fig. S2, Table S5). CAG27 negatively correlated with glucose intolerance, while CAG16 and CAG23 negatively correlated with fasting blood glucose (Fig. 4C). CAG23 had a predominant ASV from *Lactobacillus murinus*. CAG16 had ASVs from *L. johnsoni*, *Acetatifactor*, *Lachnoclostridium*, Lachnospiraceae, *Blautia*, *Roseburia*, and *Ruminiclostridium*. CAG27 primarily comprised ASVs from Lachnospiraceae, *Blautia*, *Ruminiclostridium*, *Roseburia*, and Ruminococcaceae. The relative abundance of CAG1 and CAG7 was significantly decreased in FR mice compared with FA. CAG1 and CAG7 were positively associated with the deterioration of glucose tolerance and fasting blood glucose (Fig. 4C). CAG 1 primarily consisted of ASVs from Coriobacteriaceae UCG-002 and Lachnospiraceae, while CAG7 mainly consisted of ASVs from *Fecalibaculum* and *Dubosiella*.

Collectively, these results show that our guild-based analysis identified CAGs that were both altered by TRF and associated with metabolic outcomes in HFD-fed mice.

### Time-restricted feeding conserved diurnal rhythmicity in both the whole gut microbiota and health-relevant guilds

To determine the effect of TRF on the cyclical dynamics of the gut microbiota in HFD and NFD-fed mice, we analyzed the gut microbiota structure after 11 weeks of the scheduled feeding regime. Fecal samples were collected at 6-h interval over two consecutive light–dark cycles. DNA was extracted from these samples, and the V3–V4 region of the 16S rRNA gene was sequenced. A total of 3 706 340 high-quality reads (average 30 886 ± 5365.73) were obtained and further denoised into 917 ASVs. We identified 292 out of the 293 prevalent ASVs that were previously used to construct the 34 CAGs in the week 11 microbiota data. These 292 ASVs, which accounted for ~93% of the total reads, were mapped to corresponding CAGs. We used the empirical JTK_CYCLE algorithm to detect rhythmicity in the microbial sequencing data at the CAG level. The PCoA analysis revealed a robust diurnal pattern in the overall microbial community structure in the FR and NR mice compared to their *ad libitum* counterparts (Fig. S3A and B). Consistently, the respiratory exchange ratio (RER) and energy expenditure (EE) from metabolic cage recordings also showed improved rhythmic patterns in the TRF groups (Fig. S4).

**Figure 5:**
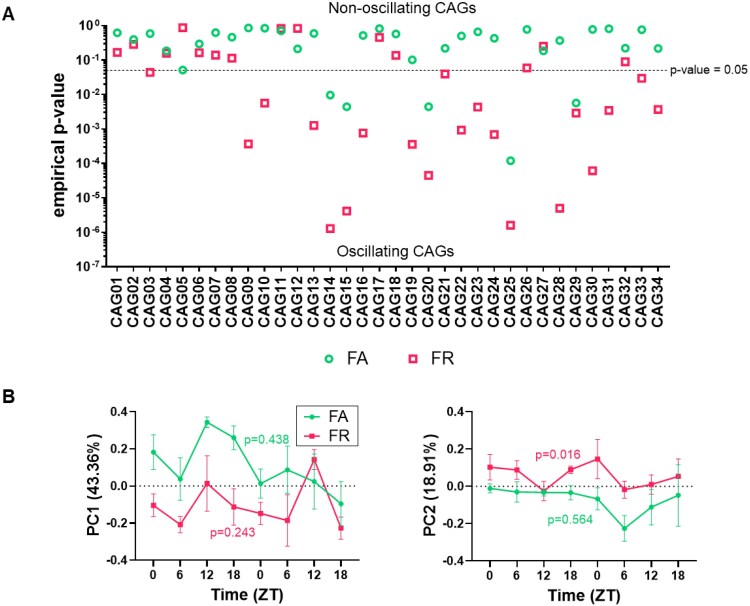
TRF displayed robust diurnal rhythmicity in gut microbiota’s overall structure and composition at the CAG level compared to mice with *ad libitum* access to HFD. (A) CAGs display diurnal oscillation in their relative abundance under TRF and *ad libitum* feeding regimes in HFD-fed mice. Rhythmicity was examined using the nonparametric empirical JTK_CYCLE algorithm, and *P*-values refer to empirical *P*-values. The dashed line indicates *P* < .05. PCoA analysis was performed at the CAG level using the abundance profile of the five health-relevant CAGs based on the Bray–Curtis dissimilarity of all the samples collected throughout two light–dark cycles during Week 11 of treatment in HFD-fed mice. (B) Alteration of the gut microbiota structure along the first and second principal coordinates (PC1 and PC2) of the PCoA based on Bray–Curtis dissimilarity in HFD-fed mice. Data were plotted as mean ± SEM; *n* = 3–5 fecal samples per time point. Rhythmicity was analyzed using the nonparametric empirical JTK_CYCLE algorithm, and *P*-values refer to empirical *P*-values.

Next, we examined whether the proportion of CAGs undergoing cyclical oscillation in relative abundance varied between feeding regimes. FR mice displayed more cyclical CAGs than FA mice (Fig. 5A, Table S6). TRF significantly increased the number of CAGs with diurnal oscillation in HFD-fed mice, from 5 in FA to 20 in FR (Fig. 5A). The NR group displayed oscillation in only 12 CAGs, far fewer than the FR group but more than the NA group with 5 CAGs undergoing diurnal oscillation (Fig. S5A, Table S7), indicating that TRF had a significant impact on the cyclical dynamics of microbial guilds in the HFD-fed mice compared to NFD-fed mice.

**Figure 6:**
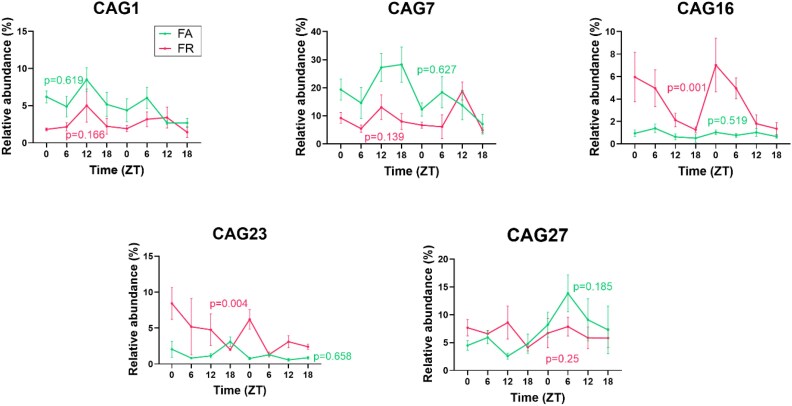
Key health-relevant CAGs associated with host metabolic parameters regained their diurnal pattern in response to TRF in HFD-fed mice. Data were plotted as mean ± SEM; *n* = 3–5 fecal samples per time point. Rhythmicity was determined using the nonparametric empirical JTK_CYCLE algorithm, and *P*-values refer to empirical *P*-values.

To determine whether health-relevant CAGs exhibited diurnal variation under TRF in HFD-fed mice, we performed PCoA on the relative abundance of the five health-relevant CAGs and applied the empirical JTK_cycle algorithm to detect rhythmicity. We observed significantly increased diurnal rhythmicity only in the FR group compared to FA along PC2 (Fig. 5B), while in NR mice, rhythmicity was not detected (Fig. S5B). Further empirical JTK_CYCLE analysis showed that two key CAGs (CAG16 and CAG23) exhibited significant diurnal oscillation in the FR group, while CAG27 remained stable. CAG1(empirical *P* = .166) and CAG7 (empirical *P* = .139), associated with impaired glucose metabolism, showed a slight trend in diurnal oscillation in FR group compared to FA group, with higher relative abundance in FA (Fig. 6).

Overall, TRF conserved diurnal rhythmicity at the CAG level including two health-relevant CAGs linked to glucose metabolism, suggesting that the conservation of microbial rhythms may contribute to the improved metabolic phenotype observed in TRF-fed HFD mice.

### Comparison of genus-based and guild-based analysis

As previous TRF studies in mice primarily used genus-based microbiome analysis, we performed a genus-level analysis on the same TRF dataset to directly compare the microbiome signatures identified by the genus- and guild-based approaches.

We conducted a comparative analysis of genus- and guild-based analysis at four levels of data reduction, using the original 1111 ASV-level data as the “ground truth” for this microbiome dataset. This included (i) Level 1: selection of ASVs for downstream analysis, (ii) Level 2: aggregation of selected ASVs into composite variables, (iii) Level 3: identification of TRF-responding composite variables, and (iv) Level 4: identification of health-relevant composite variables from the TRF-responding composite variables (Table 1, Table S8).

**Table 1:** Comparison of data reduction process between guild-based and genus-based analyses.

Data reduction method		Original dataset	Data retained	Aggregated variables	TRF-responding	Health relevant
Variable reduction	Genus-based	1111 ASVs	126 genera composed of 660ASVs	66 Genera	10 Genera	4 Genera
	Guild-based	1111 ASVs	293 ASVs	34 CAGs	7 CAGs	5 CAGs
Abundance reduction	Genus-based	2 124 000 reads	1 661 409 reads (78.22%)	1 651 349 reads (77.75%)	1 004 099 reads (47.27%)	868 790 reads (40.9%)
	Guild-based	2 124 000 reads	2 039 969 reads (96.04%)	2 039 969 reads (96.04%)	1 255 436 reads (59.11%)	1 205 732 reads (56.77%)
Sparsity reduction	Genus-based	79.92%	57.01%	23.96%	22.71%	5.72%
	Guild-based	79.92%	40.46%	2.82%	0.12%	0.17%
Level of reduction		Level 1		Level 2	Level 3	Level 4

In the genus-based approach, Level 1 reduction retained 660 out of 1111 ASVs (accounting for 78% of total reads) for further analysis, while 451 unclassifiable ASVs (22% of reads) were excluded (Table 1). In contrast, the guild-based analysis retained 293 ASVs that were present in >25% of samples, covering 96% of total reads, with only 4% excluded from downstream analysis. At level 2 reduction, the 660 classifiable ASVs in the genus-based method were aggregated into 126 genera and, after applying a prevalence cutoff of 25%, 66 genera were retained for further analysis, while the 293 prevalent ASVs in the guild-based approach were grouped into 34 CAGs via co-abundance analysis (Table 1). For level 3 reduction, random forest–based feature selection identified 10 genera and 7 CAGs as TRF-responding variables (Fig. S6A and B). At level 4 reduction, 4 genera and 5 CAGs were identified as health-relevant, as they were significantly correlated with metabolic phenotypes (Fig. S6C, Table S9, Fig. 4C, Table S5). A notable difference lies in the reduction of data sparsity. In the genus-based approach, sparsity decreased from 79.92% in the original ASV dataset to 23.96% after level 2 reduction, while the guild-based approach reduced sparsity to just 2.82% (Table 1). After level 4 reduction, sparsity remained at 5.74% for the four health-relevant genera but was reduced to 0.17% for the five health-relevant CAGs, resulting in a denser dataset in guild-based analysis that could provide more detailed insights into the microbiome–host health relationship.

We compared the ability of genus- and guild-based methods to preserve community-level information using Procrustes analysis at each level of data reduction. Procrustes analysis of PCoA plots based on the Bray–Curtis dissimilarity between the rarefied 1111 ASV dataset and the 34 CAGs before and after treatment showed a significant correlation (PROTEST analysis) (Fig. S7A and B). This indicates that, despite reducing the number of variables, CAG-level data accurately represented the overall gut microbiota structure depicted at the ASV level. Similarly, we performed PCoA and PERMANOVA analysis using Bray–Curtis dissimilarity metric to compare the abundance profiles of 66 genera with the overall gut microbiota structure from the 1111 ASVs (Fig. S8). Procrustes analysis also showed a significant correlation between the genus-level and ASV-level data (Fig. S9), but the ${m}_{12}^2$ for the genus-based analysis was higher than the guild-based approach for the baseline. For Week 12, the ${m}_{12}^2$ for genus-based analysis was lower than the guild-based approach. The information loss score, calculated as the ratio of ${m}_{12}^2$ for genus and CAG-level analysis, was 1.99 at baseline and 0.51 after 12 weeks of intervention. Comparing the 293 ASVs (grouped into 34 CAGs) vs. 1111 ASVs and 547 ASVs (classified into 66 genera) vs. 1111 ASVs, the information loss scores were undefined (${m}_{12}^2$ for 293 ASVs vs.1111 ASV = 0) and 9, respectively, at baseline and after intervention. This suggests that guild-based analysis better preserved the gut microbiota structure and minimized information loss compared to genus-level analysis.

We compared the functionally important ASVs identified by the genus- and guild-based methods. Only six ASVs (ASV001 *L. murinus*, ASV002 *Faecalibaculum*, ASV014 *L. johnsonii*, ASV12 *Blautia*, ASV107 *Blautia*, and ASV16 *Coriobacteriaceae UCG-002*) were commonly identified by both methods (Table S10). Tracking these health-relevant ASVs revealed stark differences between the two approaches. In the guild-based analysis, the five health-relevant CAGs comprised 53 ASVs, 16 of which were unclassifiable at the genus level but included due to their prevalence. These 16 ASVs, representing 113 990 reads (5.37% of total reads), were excluded from the genus-based analysis because they were unclassifiable. In contrast, the four health-relevant genera in the genus-based analysis contained 51 ASVs; 41 of these had low prevalence (below 25%) and a small number of reads (4640), accounting for only 0.2% of total reads. These low-prevalence ASVs were excluded in the guild-based analysis (Fig. S10).

In genus-based analysis, ASVs with varying responses to TRF are often aggregated into a single genus-level variable, potentially leading to spurious correlations with host health. For example, all 29 *Lactobacillus* ASVs were grouped together, resulting in an increased abundance in the FR group compared to FA mice (Fig. S11A). Further correlation analysis indicated that genus *Lactobacillus* was negatively correlated with fasting blood glucose levels, suggesting a beneficial effect. In contrast, guild-based analysis omitted 26 nonprevalent ASVs in this genus and clustered the three prevalent ones, ASV001, ASV014, and ASV105, into CAG23, CAG16, and CAG9, respectively (Figs S10 and S11B), while the abundance of CAG23 and CAG16 increased in the FR group and negatively correlated with fasting blood glucose (Fig. 4C), showing a potential beneficial effect. However, CAG9 is a nondiscriminatory CAG and is not associated with metabolic outcomes. Aggregating these ASVs with a contrasting response to TRF into a composite variable can cancel out conflicting signals, obscuring true microbial effects.

In genus-based analysis, aggregating ASVs with opposing responses to a treatment can prevent the identification of a differentially abundant genus. For example, *Roseburia,* comprising 27 ASVs, was not identified as differentially abundant between FA and FR mice (Fig. S11C). However, guild-based analysis classified 10 prevalent ASVs from *Roseburia* into seven CAGs. CAG16 (containing ASV161, ASV151, and ASV287) was negatively correlated with fasting blood glucose, and CAG27 (with ASV70) was negatively correlated with glucose intolerance. Meanwhile, ASV075 (in CAG31) abundance increased in the FA group, and the remaining ASVs showed no differential response. Thus, aggregating these ASVs at the genus level canceled out their contrasting behavior, resulting in no significant differential abundance between the groups.

## Discussion

Our study shows that restricting high-fat intake to the active phase, despite similar calorie consumption, alters the gut microbiota at the guild level, not the traditional taxon level, leading to improved body weight and glucose tolerance compared to ad libitum control. Health-relevant guilds that responded to active-phase feeding may mediate the mitigation of HFD-induced metabolic dysfunction. Additionally, TRF conserved diurnal oscillations in most health-relevant guilds, potentially preventing metabolic deterioration seen with *ad libitum* HFD feeding.

Previous studies suggest that TRF’s metabolic benefits are proportional to fasting duration, but extended fasting often results in lower caloric intake [1, 10, 32], which may confound the effects of TRF. Reduced calorie intake significantly modulated the gut microbiota [33], making it difficult to isolate the impact of TRF alone in those studies. In contrast, our study controlled for calorie intake with isocaloric TRF, allowing us to attribute the observed benefits to the gut microbiota response rather than reduced caloric intake.

In this study, TRF-induced changes in five CAGs were associated with metabolic outcomes in HFD-fed mice. Three guilds (CAG16, CAG23, and CAG27) responded positively to TRF on HFD and were negatively associated with disease parameters, suggesting they may be beneficial. CAG23 included *L. murinus* (ASV1), which has been linked to improved metabolic phenotypes in mice [33]. CAG16 contained *L. johnsonii* (ASV14), with some strains exhibiting anti-inflammatory effects and the ability to inhibit gut pathogens in mice [34, 35]. Some *L. johnsonii* strains also improve insulin secretion in rodents [36]. Additionally, CAG16 included members from Lachnospiraceae family, which produce short-chain fatty acids (SCFAs) and bacteriocins, contributing to metabolic health [37, 38]. CAG27 was dominated by members of *Lachnospiraceae*, including both unclassified members as well as species from *Blautia* and *Roseburia.* While *Blautia* species are linked to improved glucose intolerance and increased SCFA production [39], a prior TRF study did not find *Blautia* to be increased in response to TRF [2]. Although Lachnospiraceae can be implicated in metabolic dysfunction [37], their presence in a health-relevant CAG suggests that they form part of a beneficial guild. Conversely, CAG7, primarily composed of *Faecalibaculum* and *Dubosiella* ASVs, correlated with impaired glucose tolerance and elevated glycemic levels. *Faecalibaculum* has been associated with weight gain and glucose intolerance [40]. TRF increased the *Dubosiella* genus in [2], though strain-level variations may influence its response [3, 41]. CAG1 exhibited a similar response to CAG7 and is primarily composed of *Coriobacteriaceae* UCG-002, which has been linked to intestinal inflammation [42], suggesting these may represent pathobiont guilds. The guild-based approach clusters known and unknown bacteria into functional groups, helping to identify novel beneficial or pathogenic bacteria based on their prior guild associations.

In this study, we demonstrate that TRF promotes daily oscillations in the overall gut microbiota structure at the guild level. Additionally, two key CAGs associated with improved glycemic control in HFD-fed mice conserved diurnal rhythms in response to TRF. For example, CAG23 and CAG16, containing *Lactobacillus* members, exhibited cyclical oscillation under TRF. Prior studies report similar oscillations in the Lactobacillus genus, despite its abundance being linked to both detrimental [1] and beneficial [2] metabolic effects. This indicates that both the differential abundance and diurnal oscillatory behavior of key guilds play a role in mediating TRF’s beneficial effects in HFD-fed mice.

However, the mechanisms linking these diurnal microbial oscillations and metabolic health need further study. Niche modification, where initial bacterial activity alters the environment to support the growth of other bacteria, may drive these diurnal dynamics [43]. For example, CAG23 showed significant oscillation only in the FR group, while nonoscillating guilds like CAG27 remained stable. TRF-induced cyclical oscillations may also conserve cyclical oscillation in microbially derived metabolites, such as SCFAs [12], which can influence host metabolism.

A unique aspect of this study is the systematic comparison of guild- and genus-based analyses using the same TRF microbiome dataset. While both methods reduce data complexity, the guild-based approach preserved the gut microbiome’s overall structure more effectively with fewer aggregated variables and reduced dataset sparsity. This higher matrix density improves the reliability of statistical inferences about microbiome–host health relationships [44, 45]. Only six ASVs were identified as health-relevant by both methods, highlighting their conceptual differences. The genus-based approach focuses on predominantly identified taxa, potentially missing complex microbial interactions. In contrast, the guild-based analysis offers a more holistic view, unconstrained by taxonomic limitations, providing deeper insights into the gut microbiome's functional dynamics under TRF.

Our study highlights key limitations of genus- or taxon-based analysis: (i) Exclusion of novel microbes: ASVs that cannot be classified at the genus level are excluded, potentially omitting important, understudied microbes. For example, ASV007 from *Lachnospiraceae*, one of the top 10 ASVs in the dataset, was excluded in the genus-based approach. This underscores the risk of overlooking predominant but poorly characterized community members. (ii) Aggregation leading to misrepresentation: Aggregating ASVs within the same genus, despite their diverse ecological behaviors, can produce misleading results. This often leads to inconsistent correlations of the same taxon with disease across different studies, manifesting as no correlation, positive correlation, or negative correlation [46, 47]. Specifically, *Lactobacillus* frequently exhibits discrepancies in its association with metabolic outcomes related to type 2 diabetes [48]. In our study, the guild-based approach identified three *Lactobacillus* ASVs, each with contrasting responses to TRF: two were potentially beneficial, while ASV105 emerged as nonresponsive. Lumping all *Lactobacillus* ASVs into a single variable could mask these differences. The predominant ASVs may dictate the genus-level outcome, obscuring the contributions of functionally significant but less abundant ASVs, leading to conflicting findings reported by different studies [1, 2]. In contrast, the guild-based strategy groups prevalent ASVs based on co-abundance pattern, independent of taxonomy. This unsupervised, database-independent method allows for the identification of health-relevant guilds while preserving detailed ASV-level information.

There are limitations to the current study; first, the use of only male mice may overlook sex-specific differences in responses to TRF. Female mice may exhibit distinct metabolic and microbiome-related adaptations that are not represented in these findings [49, 50]. Including female mice in future studies will be important to fully assess sex-specific effects of TRF. Second, the lack of measurements of microbial metabolites, such as SCFAs, limited our ability to explore how changes in microbiota composition relate to functional metabolic outcomes under TRF.

Our study shows that TRF alleviates HFD-induced weight gain and glucose intolerance, associated with specific gut microbiota changes at the guild level, despite similar calorie intake. Moreover, two responding bacterial guilds display diurnal oscillating patterns under TRF in HFD-fed mice. These alterations in the gut microbiota may contribute to reducing the adverse metabolic effects of HFD. Further research is needed to elucidate the mechanisms through which TRF-enriched bacterial guilds contribute to these benefits. Moreover, while genus- or taxon-based analyses may overlook important microbes and aggregate ASVs with different ecological behaviors within the same genus into the new variable, our guild-based approach, which groups ASVs based on co-abundance patterns rather than taxonomy, offers a more accurate representation of microbial diversity and its associations with health outcomes. This method enables the identification of health-relevant guilds without masking subtle, yet significant, ASV-level differences.

## Supplementary Material

TRF_supplementalfigures_SG_ISMECOMM_F1_ycaf127

TRF_supplementary_Tables_ISME_F1_ycaf127

## Data Availability

All data associated with this study are included in the manuscript or the supplementary materials. No custom code was used in this study. The 16S rRNA gene sequence data generated in this study has been submitted to Sequence Read Archive (SRA) maintained by NCBI under the accession number PRJNA975214. Reviewer link: https://dataview.ncbi.nlm.nih.gov/object/PRJNA975214?reviewer=fdgi3806rv3b0nv8n78p2elq9a
