# Supplementary material for: Bacterial guilds, not genus-level taxa, mediate the protective effects of time-restricted feeding against high-fat diet–induced obesity in mice
Source: ISME Commun. 2025 Oct 11;5(1):ycaf127. doi: 10.1093/ismeco/ycaf127 (PMC12515041; doi:10.1093/ismeco/ycaf127)
Supplement: TRF_supplementalfigures_SG_ISMECOMM_F1_ycaf127 [file trf_supplementalfigures_sg_ismecomm_f1_ycaf127.pdf]

## Supplementary Figures

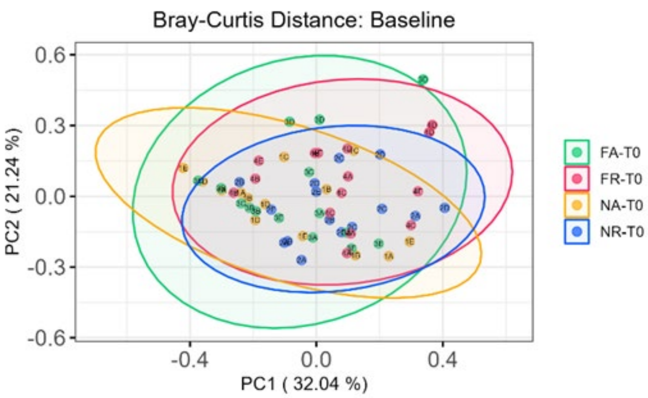


Figure S1. PCoA based on the Bray-Curtis dissimilarity metric of the rarefied baseline ASV dataset (1,111 ASVs), with the cage identities overlaid.

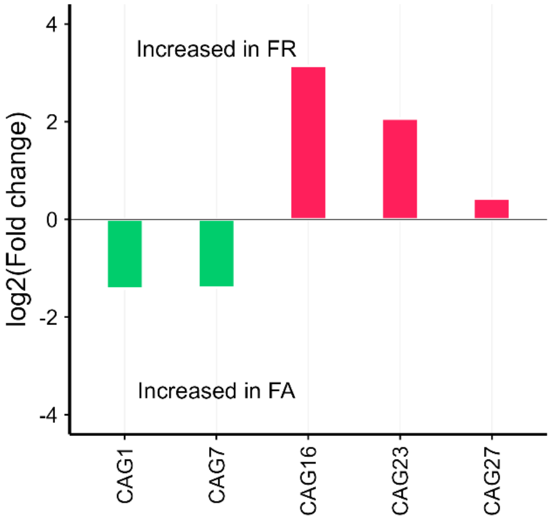


Figure S2. Relative abundance of the key CAGs. Barplot of the Log2 fold change in relative abundance of the key CAGs

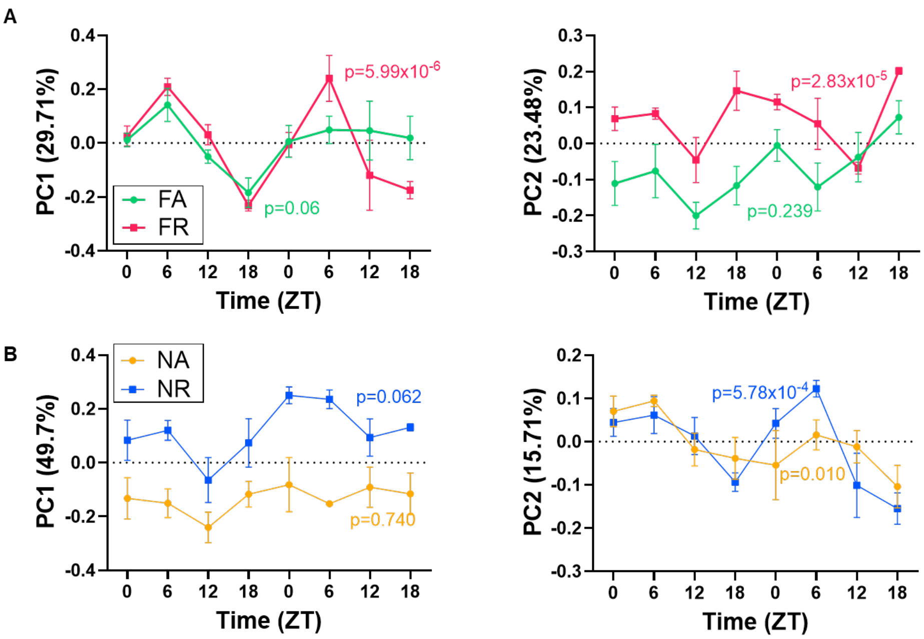


Figure S3. TRF displayed robust diurnal rhythmicity in gut microbiota's overall structure and composition at the CAG level compared to mice with *ad libitum* access to diet. PCoA analysis was performed at the CAG level using the abundance profile of the 34 CAGs based on the Bray-Curtis dissimilarity of all the samples collected throughout two light-dark cycles during week-11 of treatment in HFD-fed mice. Alteration of the gut microbiota structure along the first and second principal coordinate (PC1 and PC2) of the PCoA based on Bray-Curtis dissimilarity in (A) HFD-fed mice and (B) NFD-fed mice. Data were plotted as mean ± SEM.; n=3-5 fecal samples per time point. Rhythmicity was analyzed using the non-parametric empirical JTK_CYCLE algorithm and p-values refer to empirical p-values.

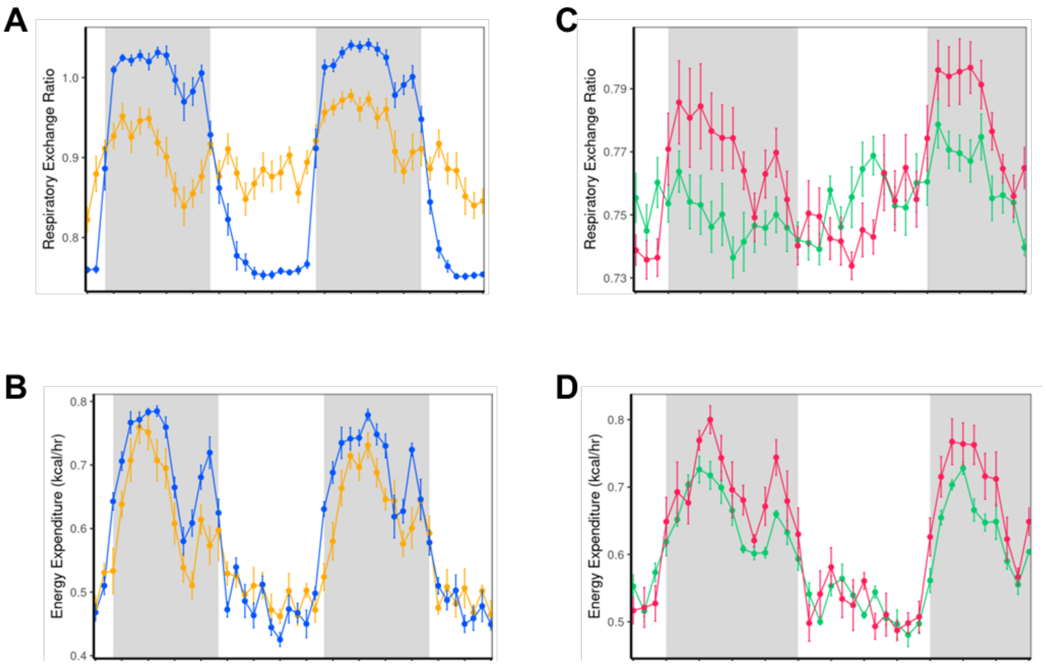


Figure S4. Respiratory exchange ratio (RER) and energy expenditure (EE) in NFD- and HFD-fed mice. RER and EE from metabolic cages recordings in (A, B) NFD-fed mice and (C, D) HFD-fed mice after 12 weeks of dietary intervention (n=5/group). The gray color indicates the active phase, and the white color indicates the rest phase.

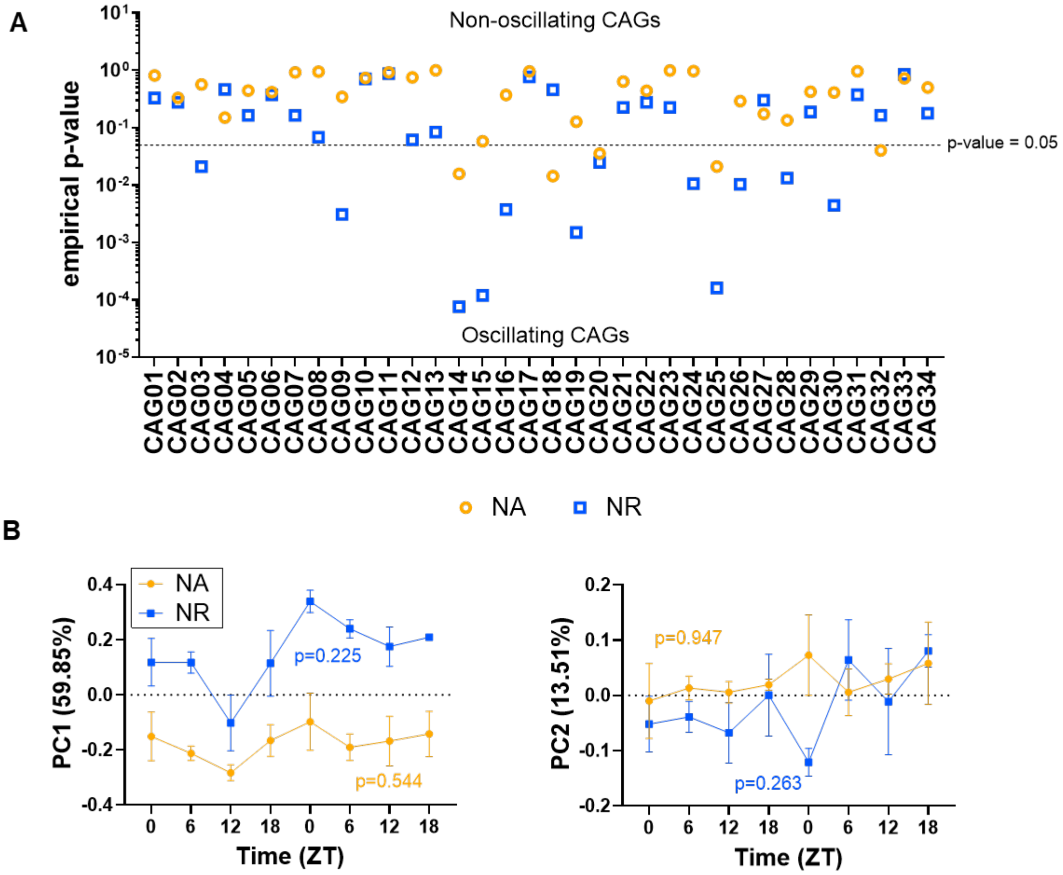


Figure S5. TRF displayed increased diurnal rhythmicity in the overall structure and composition of gut microbiota compared to mice with ad libitum access to NFD at the CAG level. (A) CAGs display diurnal oscillation in their relative abundance under NA and NR feeding regime. Rhythmicity was examined using the non-parametric empirical JTK-cycle algorithm and p-values refer to empirical-p values. The dashed line indicates p=0.05. PCoA analysis was performed at the CAG level using the abundance profile of the 5 key CAGs based on the Bray-Curtis dissimilarity of all the samples collected over the course of two light-dark cycles during week-11 of treatment. (B) Alteration of the gut microbiota structure along the first and second principal coordinate (PC1 and PC2) of the PCoA based on Bray-Curtis dissimilarity. Data were plotted as mean ± s.e.m.; n=3-5 fecal samples per time point. Rhythmicity was analyzed using the non-parametric empirical JTK-cycle algorithm and p-values refer to empirical-p values.

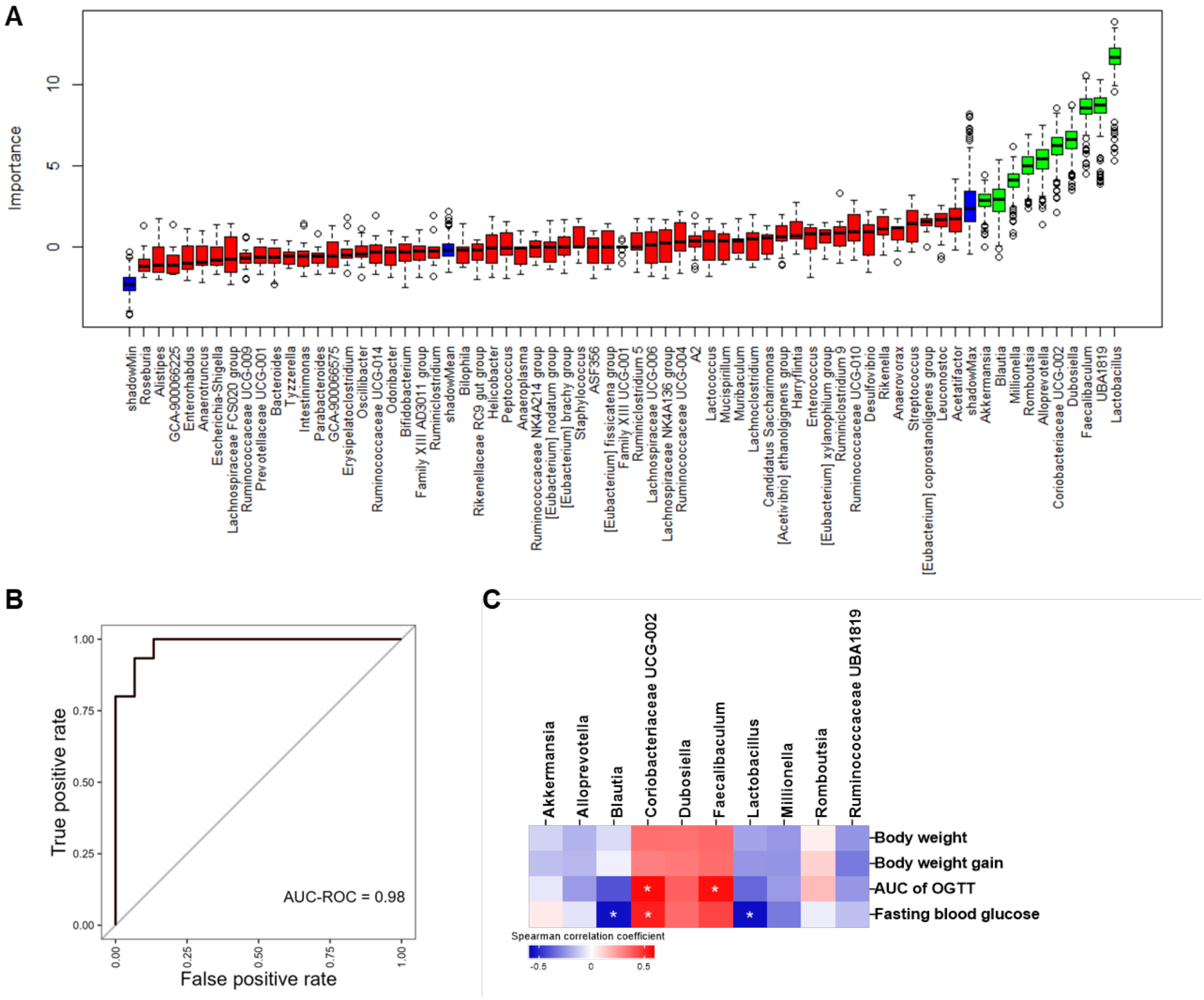
A
Importance
B
True positive rate
False positive rate
AUC-ROC = 0.98
C
Body weight
Body weight gain
AUC of OGTT
Fasting blood glucose
Spearman correlation coefficient


706

Figure S6. TRF-responding genera identified by Boruta, a random forest-based feature selection method. (A) Boxplot of the importance score (Z score) of the CAGs identified by Boruta for differentiating between FA and FR after 12 weeks of intervention. The boxplots in “green” were identified as key variables capable of discriminating between FA and FR, whereas the boxplots in “red” were found as non-discriminatory. (B) The area under the ROC curve for the genera-based classification of the FA and FR group (AUC=0.980, 95% CI 0.93 -1.03); ROC: Receiver operating characteristic. (C) Heatmap of Spearman’s correlation (with FDR correction) between the relative abundance of the discriminating genera identified by Boruta, and the metabolic parameters related to glucose and lipid metabolism. *$p < 0.05$, and **$p < 0.01$.

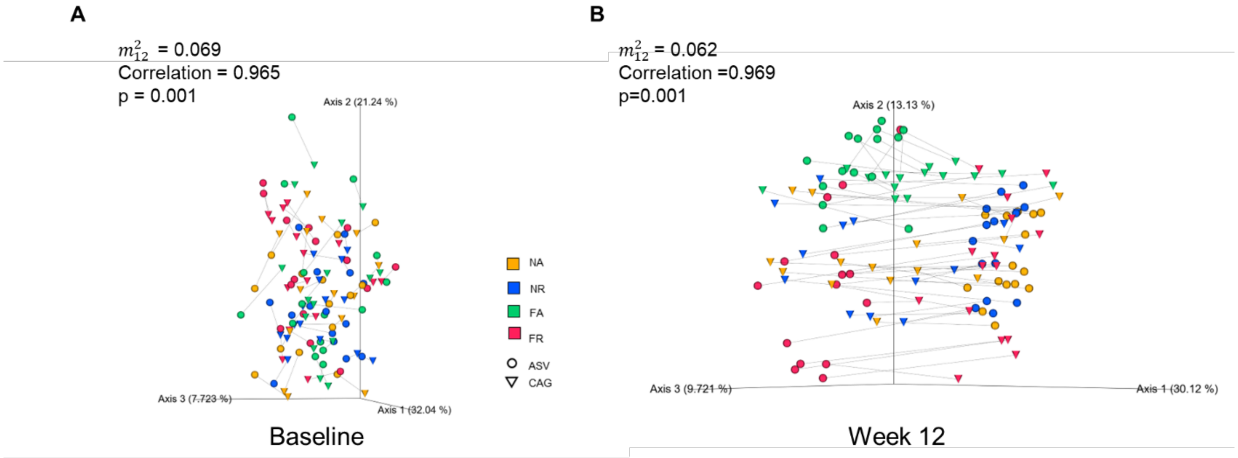


Figure S7. Concordance between the gut microbiota structure at ASV and CAG-levels. Procrustes analysis was performed on the PCoA based on the Bray-Curtis dissimilarity metric for the rarefied ASV dataset and the PCoA on the relative abundance profiles for the 34 CAGs based on the Bray-Curtis dissimilarity metric at (A) baseline and (B) after implementing 12 weeks of TRF regime. PROTEST analysis was used to test for statistical significance between the two ordinations with 999 permutations.

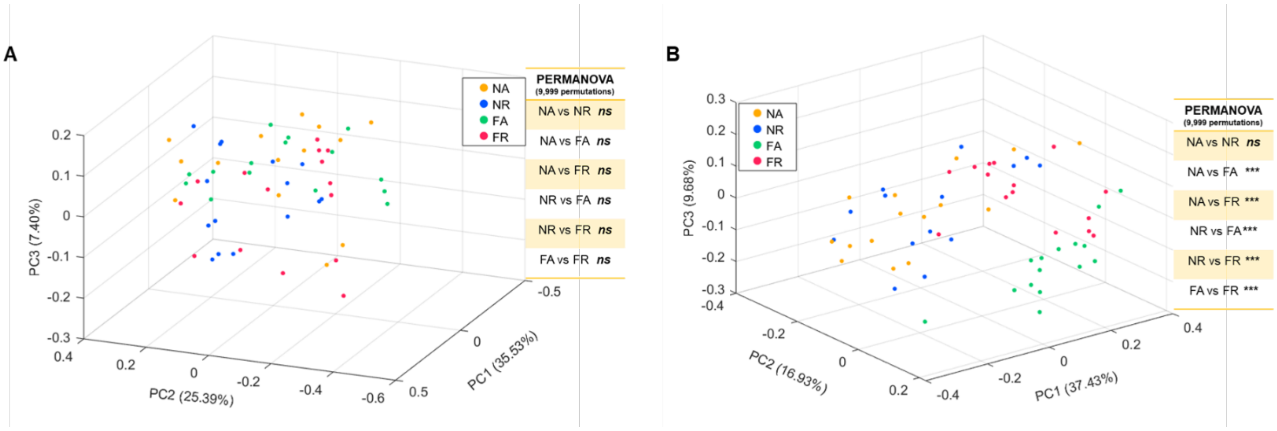


Figure S8. TRF altered the gut microbiota structure at the genus-level in HFD-fed mice. Principal-coordinate analysis (PCoA) was performed using the relative abundance profile at the genus-level with 66 genera based on the Bray-Curtis dissimilarity metric at (A) baseline and (B) after 12 weeks

of TRF regime.

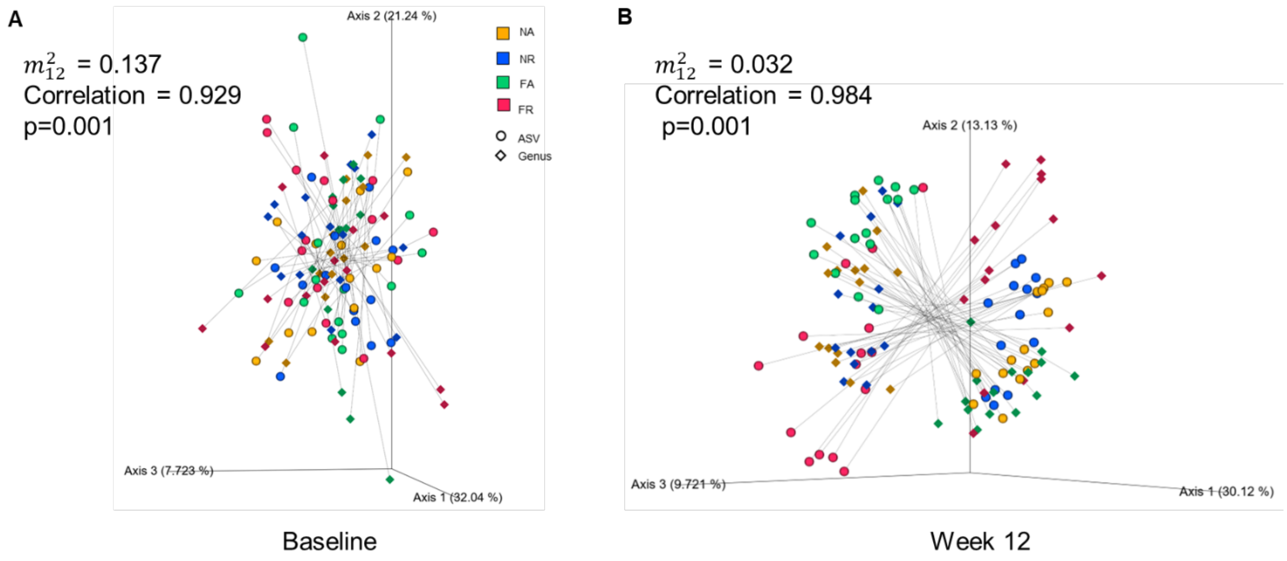


Figure S9. Concordance between the gut microbiota structure at ASV and genus levels. Procrustes analysis was performed on the Bray-Curtis dissimilarity PCoA plots derived from the rarefied ASV dataset and the relative abundance profiles for the 130 genera at (A) baseline and (B) after implementing 12 weeks of TRF regime. PROTEST analysis was used to test for statistical significance between the two ordinations with 999 permutations.

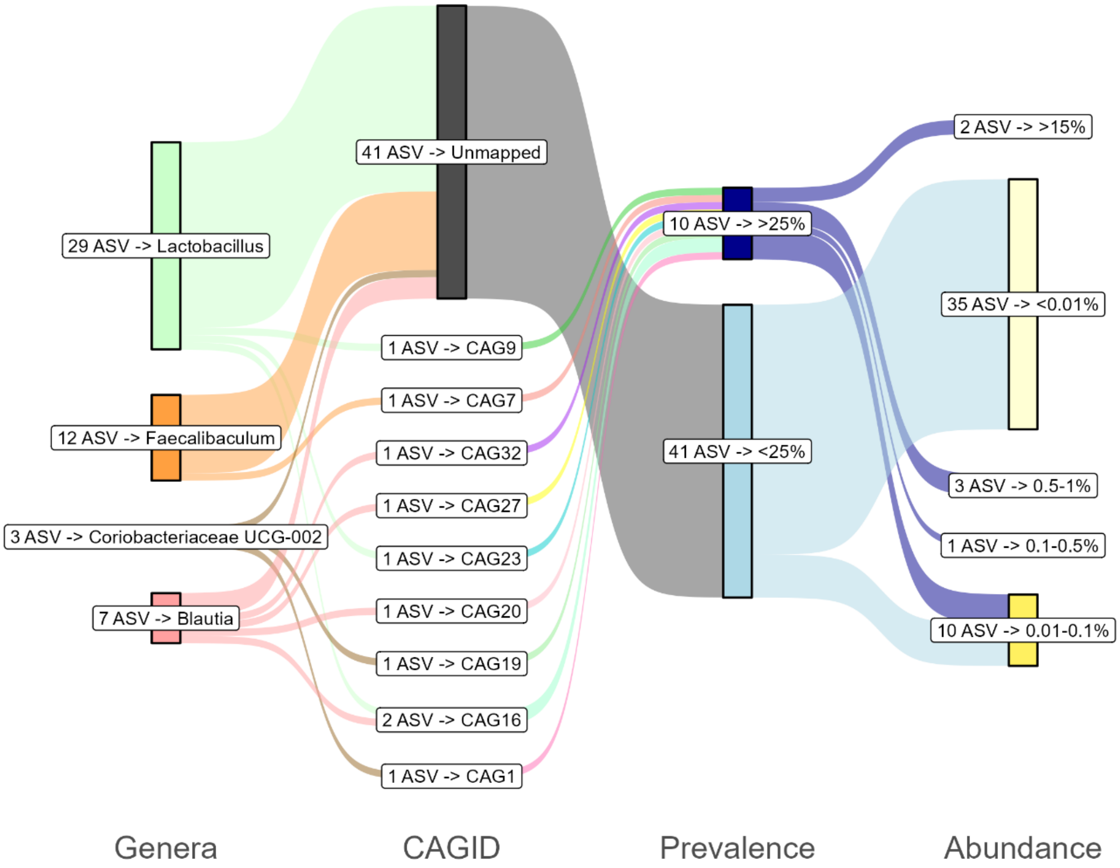


Figure S10. Most of the ASVs identified as part of health-relevant genera had low prevalence and abundance. Sankey diagram reveals that out of 51 ASVs identified as part of health-relevant genera, only 10 could be mapped to CAGs. The remaining 41 ASVs could not be mapped to a CAG. This is because the prevalence of each of these ASVs was less than the prevalence cutoff of 25%, which was used in the guild-based analysis.

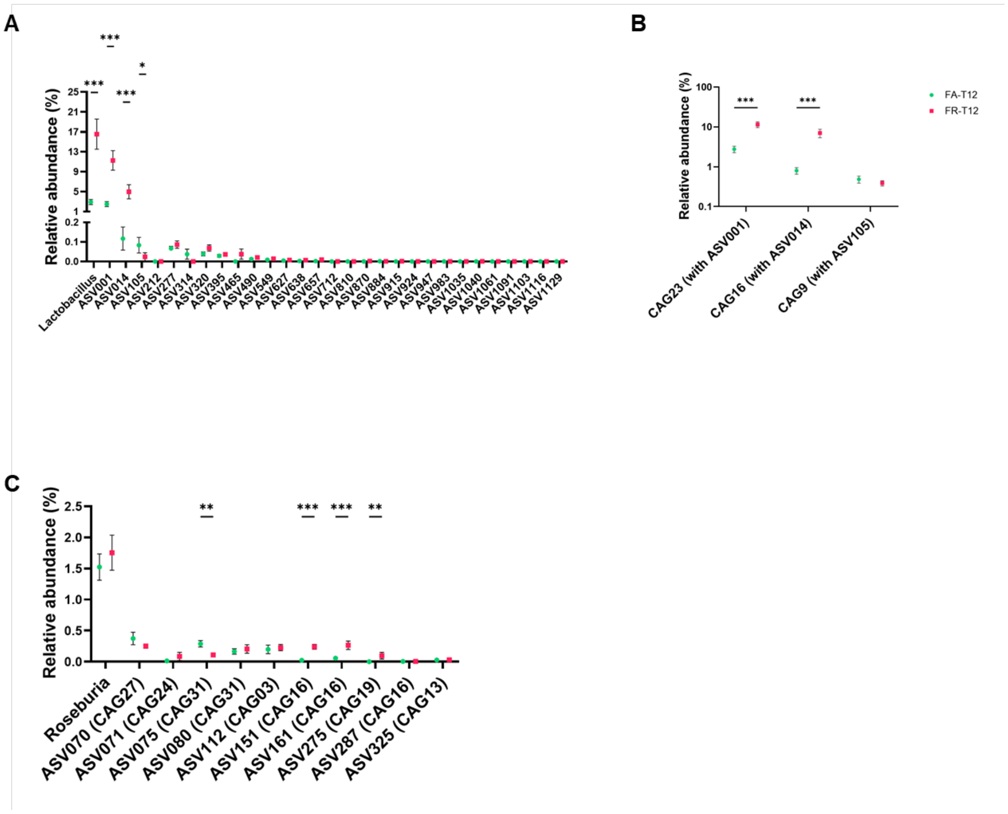


Figure S11. Comparison between Genus and CAG-based methods. CAG-based analysis captures the differential response of the individual ASVs accurately. (A) shows the distribution of abundance of the twenty-eight individual ASVs that were classified as *Lactobacillus* genus in the FA and FR groups. In taxon-based analysis all 29 ASVs will be aggregated together, and their total abundance is presented as *Lactobacillus* genus. (B) shows the distribution of the 3 CAGs that contain the 3 differentially abundant ASVs from *Lactobacillus* genus. In CAG-based analysis, the three differentially abundant ASVs were grouped in different CAGs based on their co-abundance pattern. It highlights that the ASV001 and ASV014 were increased in FR and are part of CAG23 and CAG16 respectively while the ASV105 that is reduced in FR is part of CAG9. (C) shows the abundance of *Roseburia* genus with 27 ASVs along with 10 ASVs from *Roseburia* genus that were mapped to a CAG. The 10 ASVs are part of different CAGs which is indicated next to each ASV on the plot. The symbols in a and b represent the mean abundance and differential abundance between FA and FR group was tested by Mann-Whitney test.
